# Chikungunya Virus Infection Results in Higher and Persistent Viral Replication in Aged Rhesus Macaques Due to Defects in Anti-Viral Immunity

**DOI:** 10.1371/journal.pntd.0002343

**Published:** 2013-07-25

**Authors:** Ilhem Messaoudi, Jennifer Vomaske, Thomas Totonchy, Craig N. Kreklywich, Kristen Haberthur, Laura Springgay, James D. Brien, Michael S. Diamond, Victor R. DeFilippis, Daniel N. Streblow

**Affiliations:** 1 Vaccine and Gene Therapy Institute, Oregon Health and Science University, Beaverton, Oregon, United States of America; 2 Molecular Microbiology and Immunology, Oregon Health and Science University, Portland, Oregon, United States of America; 3 Division of Pathobiology and Immunology, Oregon National Primate Research Center, Beaverton, Oregon, United States of America; 4 Departments of Molecular Microbiology, Medicine, Pathology and Immunology, Washington University School of Medicine, St. Louis, Missouri, United States of America; University of Texas Medical Branch, United States of America

## Abstract

Chikungunya virus (CHIKV) is a re-emerging mosquito-borne Alphavirus that causes a clinical disease involving fever, myalgia, nausea and rash. The distinguishing feature of CHIKV infection is the severe debilitating poly-arthralgia that may persist for several months after viral clearance. Since its re-emergence in 2004, CHIKV has spread from the Indian Ocean region to new locations including metropolitan Europe, Japan, and even the United States. The risk of importing CHIKV to new areas of the world is increasing due to high levels of viremia in infected individuals as well as the recent adaptation of the virus to the mosquito species Aedes albopictus. CHIKV re-emergence is also associated with new clinical complications including severe morbidity and, for the first time, mortality. In this study, we characterized disease progression and host immune responses in adult and aged Rhesus macaques infected with either the recent CHIKV outbreak strain La Reunion (LR) or the West African strain 37997. Our results indicate that following intravenous infection and regardless of the virus used, Rhesus macaques become viremic between days 1–5 post infection. While adult animals are able to control viral infection, aged animals show persistent virus in the spleen. Virus-specific T cell responses in the aged animals were reduced compared to adult animals and the B cell responses were also delayed and reduced in aged animals. Interestingly, regardless of age, T cell and antibody responses were more robust in animals infected with LR compared to 37997 CHIKV strain. Taken together these data suggest that the reduced immune responses in the aged animals promotes long-term virus persistence in CHIKV-LR infected Rhesus monkeys.

## Author Summary

Chikungunya virus (CHIKV) is a re-emerging Alphavirus that has caused recent massive outbreaks in the Indian Ocean region. In addition, outbreaks have been documented in Europe and elsewhere in the world, initiated by infected travelers returning to their homelands. The recent outbreak strains possess extended vector range and as such, raise the potential of CHIKV outbreaks in the Southeastern parts of the United States. In this study, we examined CHIKV immunity in adult and aged Rhesus macaques following infection with two different CHIKV strains (recent outbreak strain CHIKV-LR and a West African Strain CHIKV-37997). CHIKV-LR causes persistent infection in the aged animals and replicates, on average, to higher levels than CHIKV-37997. Irrespective of the viral strain used, aged animals had delayed and/or reduced immunity compared to adult animals. Our data support the clinical findings of CHIKV susceptibility in vulnerable populations including the aged and provide mechanistic evidence that an effective immune response directed against the virus is required for preventing persistent CHIKV infection.

## Introduction

Chikungunya virus (CHIKV) is a re-emerging member of the Alphavirus genus within the Togaviridae family. CHIKV was first isolated in the 1950's from the serum of a febrile patient in Tanzania during a dengue fever-like outbreak [1]. The virus and the associated disease were named chikungunya, meaning “that which bends up” in the local language in reference to the debilitating poly-arthralgia that accompanies the infection [2]. Minor outbreaks of CHIKV continued to occur until 2004 when a large epidemic in Kenya marked the beginning of a 4-year period in which CHIKV was imported into several new countries [3], [4]. Several outbreaks occurred in India, where over one million cases were reported [5], [6]. In 2006, over 200,000 cases were reported on the island of La Reunion [7], [8], the most significant aspect of that outbreak being that a single point mutation in the viral envelope glycoprotein allowed the virus to replicate to very high titers in both *Aedes (Ae.) albopictus* and *Ae. aegypti* mosquitos, which are widely distributed throughout the world [3], [9], [10]. Viremic travelers returning from India initiated a local outbreak in Italy through infection of *Ae. albopictus* mosquitoes [11], [12]. CHIKV cases from travelers returning from endemic regions were also reported in France and the United States, demonstrating the potential of CHIKV spread to distant locales [13], [14].

Clinical symptoms of CHIKV infection in humans include acute fever lasting up to two weeks and severe poly-arthralgia of the peripheral joints that can be very debilitating and last for several months. Additional symptoms include nausea, headache, rash and lymphadenopathy. Although CHIKV infection is usually a self-limiting disease, the outbreaks occurring after 2005 were explosive and exhibited complicated clinical-pathological manifestations including neurological involvement. Aged individuals and adults with underlying immunological conditions experienced severe morbidity and sometimes mortality [15] indicating that a functional immune system maybe important to control infection and promote recovery. Recent epidemics also showed the first CHIKV intra-partum maternal–fetal transmission with devastating outcomes [16]. In addition, age-matched studies during an outbreak in Singapore found that women are more susceptible to CHIKV-induced chronic arthralgia than men [17], [18]. Treatment of CHIKV infection and disease is currently limited to supportive care. Therefore, the development of effective CHIKV vaccines and therapeutics are currently being intensely explored.

The immune response to CHIKV infection has been relatively understudied. A limited number of studies characterized changes in plasma cytokine/chemokine levels following CHIKV infection in order to identify predictors of poor disease prognosis. These analyses revealed that the acute phase of infection (4 days post onset of illness) is associated with the production of robust Type-1 interferon (IFN) [17], [19]. The level of IFN directly correlated with viral load [20]. The chemokines IP-10 (CXCL10), MCP-1 (CCL2) and RANTES as well as the cytokines IL-6, IL-1β IL-1Rα and IL-12 are similarly expressed early following infection and their levels correlate with viral loads [21]. Similar findings have been observed in CHIKV-infected non-human primates [22]. High viral loads during the early viral convalescence phase have also been associated with increased IL-12 and IL-6 levels compared to patients with lower viral loads [17]. Interestingly, increased IL-6 and GM-CSF are observed in patients experiencing chronic joint pain (2–3 months post onset of disease) whereas, increases in Eotaxin and hepatocyte growth factors are associated with a full recovery from CHIKV disease [17]. The functional relationship between expression of these two proteins and disease recovery warrants further investigation.

The adaptive immune responses to CHIKV infection have not been as widely investigated. In one study, the acute phase of CHIKV infection in a human cohort from a 2007 CHIKV outbreak in Gabon was found to be associated with CD8+ T cell activation. However, the antiviral specificity of the CD8 T cell response and its contribution to disease resolution were not investigated [19]. CHIKV-associated antibody responses have been better characterized. CHIKV infection induces robust neutralizing antibody responses in both humans and animal models. In fact, neutralizing antibodies directed against E2 have been found to be protective and the early presence of IgG3 antibodies have been correlated with protection from persistent arthritis in patients from a 2008 CHIKV outbreak in Singapore [23], [24]. Unfortunately, we have a limited knowledge as to the viral protein(s) are targeted or by what specific immune cell subsets, nor do we know the timing of these events. Here, we describe the infection of adult and aged Rhesus macaques with either the Senegal strain (CHIKV-37997) or the reemergent strain (CHIKV-LR_Opy-1_). We detail the innate and adaptive immune antiviral responses following infection. Rhesus macaques infected with CHIKV become viremic within 24 hours of inoculation and this was typically resolved by 5 days post-infection (dpi). All animals generated robust T and B cell responses that peaked by 14 dpi. However, our study demonstrates that aged animals persistent viral RNA in the spleen. Interestingly, this inability to clear the virus in the aged animals correlated with reduced innate and adaptive immune responses compared to adult animals. Our findings suggest that defects in the aged immune system play a critical role promoting CHIKV persistence.

## Materials and Methods

### Ethics statement

All Rhesus macaques were handled in accordance with good animal practice as defined by relevant national and/or local animal welfare bodies. The use of non-human primates was approved by the Oregon National Primate Research Center (ONPRC) Institutional Animal Care and Use Committee (IACUC #0826). The ONPRC is fully accredited by the Assessment and Accrediatation of Laboratory Animal Care-International. For blood collection monkeys were anesthetized with ketamine by intramuscular injection. Monkeys were humanely euthanized by the veterinary staff at ONPRC in accordance with endpoint policies. Euthanasia was conducted under anesthesia with ketamine followed by overdose with sodium pentobarbital. This method is consistent with the recommendation of the American Veterinary Medical Association.

### Viruses

The full-length infectious clones for the West African (Senegal) strain CHIKV-39779 and the recent outbreak strain La Réunion (LR2006 OPY1; CHIKV-LR) were obtained from Dr. Stephen Higgs University of Texas Medical Branch-Galveston. The viruses produced from the infectious clones retain their original viral phenotypes of CHIKV in both cell culture and in mosquitoes. RNA was transcribed using the T7 mMessage mMachine kit (Ambion). The transcribed RNA was transfected into BHK cells and the resultant virus was propagated in C6/36-insect cells to produce low passage, high titer CHIKV stocks by pelleting through a 20% sucrose cushion by ultracentrifugation (22,000 rpm, 82520×g for 1.5 hrs). Plaque titration assays on Vero cells using a carboxy-methylcellulose overlay were performed to determine titers (plaque forming units) of the stock virus.

RNA transcribed from the infectious clones of CHIKV-LR and CHIKV-37997 was transfected into BHK cells and passaged one time in insect cells to generate the virus stock used to infect the animals.

### Animal infection and sample collection

Adult male and female rhesus macaques (ranging from 6–13 years old) and aged female rhesus macaques (>17 years old) were utilized for infection studies as described in Table 1. Animals were infected intravenously with 1×10^7^, 1×10^8^ or 1×10^9^ plaque forming units (pfu) CHIKV-LR or CHIKV-37997 strain in 1 ml of phosphate buffered saline (PBS), and a sample of all viral inoculums was back-titered to confirm the delivery of correct dosage. Blood samples were obtained following ketamine sedation (10 mg/Kg) at 4 and 2 weeks prior to infection, on the day of infection (day 0), 1, 2, 3, 5, 7, 10 and14 days post infection and on a weekly basis thereafter. Complete blood cell counts were obtained at every time point to monitor changes in immune cell numbers/µl of blood. Peripheral blood mononuclear cells (PBMC) were isolated from whole blood by centrifugation over Histopaque gradient (Histopaque, Sigma-Aldrich, St Louis, MO).

**Table 1:** Animal groupings.

ID	Groups	Sex	Wt (kg)	Age (Y)	CHIKV Strain	Virus Dosage [36]
20374	1/A	F	6.4	11	LR	1×10^7^
21384	1/A	F	5.8	13	LR	1×10^7^
21551	1/B	F	6.6	9	LR	1×10^8^
22477	1/B	F	6.2	8	LR	1×10^8^
23643	1/C	M	13.3	6	LR	1×10^9^
28525	1/C	M	12.2	10	LR	1×10^9^
20969	1/aged	F	7	18	LR	1×10^9^
22163	1/aged	F	6.2	18	LR	1×10^9^
22855	2/A	F	5.6	11	37997	1×10^7^
22865	2/A	F	7.2	13	37997	1×10^7^
22910	2/B	F	5.4	11	37997	1×10^8^
23465	2/B	F	8.2	11	37997	1×10^8^
28524	2/C	M	13.5	8	37997	1×10^9^
28520	2/C	M	8.4	9	37997	1×10^9^
20841	2/aged	F	6.2	17/325	37997	1×10^9^
20994	2/aged	F	6.4	18	37997	1×10^9^

### Quantitative RT-PCR analysis for CHIKV

Viral load in peripheral blood (plasma and PBMC) as well as tissues at necropsy of the rhesus macaques was quantified using real-time RT-PCR. Primers and probes include: CHIKV-LR fwd: GGAACGAGCAGCAACCTTTG, rev: ATGGTAAGAGTCTCAGACAGTTGCA, and probe: GGAATAAGGGCTTGT; CHIKV-37997 fwd: CGGAGAGCCGTAAGCTTCTTAA, rev: TTCACACGAAACCACTGTATCACA, and probe: CTTCAGTGTTCCATCTAAA. Total RNA was prepared from 100 µl of animal sera using the ZR Viral RNA kit (Zymo Research, Irvine, CA) or from 5×10^6^ total PBMC using Trizol. RNA was prepared from tissue specimens by the Trizol method. The isolated RNA was quantified using a Nanodrop spectrophotometer. RNA was first treated with RNAse-free DNAse and then single stranded cDNA was generated using random hexamers and Superscript III RT (Invitrogen, Carlsbad, CA). Gene amplicons served as quantification standards (limit of detection is 10–100 copies). Quantiative RT-PCR results were analyzed using ABI StepOne Plus Real-Time PCR system (Applied Biosystems, Foster City, CA).

### CHIKV overlapping peptide library

CHIKV peptides were designed to be 18 mers that overlapped by 12 amino acids covering the entire CHIKV proteome (peptides were purchased from Thermo Fischer Scientific). Peptides were resuspended in DMSO at 1 mg/ml. Separate peptides pools were made for each of the nine viral protein products.

### Measuring CHIKV-specific T cell responses

PBMC were stained with surface antibodies against CD4, CD8β, CD28, and CD95 to delineate naïve, central memory (CM), and effector memory (EM) T cell subsets. All antibodies with the exception of CD8β (Beckman Coulter) were purchased from Biolegend (San Diego, CA). Cells were then fixed and the nuclear membrane permeabilized as per manufacturer's recommendation (BD Pharmingen, San Diego, CA) before staining with anti-Ki67 (BD Pharmingen, San Diego, CA). Samples were acquired using the LSRII instrument (BD bioscience, San Jose, CA) and data analyzed by FlowJo (TreeStar, Ashland, OR).

Gamma interferon (IFN-γ) enzyme linked immunospot (ELISPOT) assays were performed using the ELISpotPlus for Monkey IFN-γ kit (MabTech). Plates were washed one time with 200 µl/well of PBS and then blocked for 30 minutes in 200 µl/well of RPMI containing 10% fetal calf serum (FCS) and penicillin, streptomycin and glutamine (RPMI-10). Overlapping CHIKV peptide pools (1 µg/well) for each of the nine CHIKV proteins were added to wells containing 2×10^5^ rhesus monkey PBMC (n = 3). Positive controls (1 µl of each phorbol 12-myristate 13-acetate (PMA; 200 mg/ml stock) and Ionomycin (7 mM stock)) and negative controls (DMSO; 1 µl) were also performed for each monkey PBMC sample. Plates were incubated for 18 hrs at 37°C. After incubation the plates were washed thoroughly with PBS and then incubated with a biotin-conjugated anti-IFN-γ antibody (1 µg/ml in PBS plus 0.5% FCS) for 2 hrs at room temperature. Plates were washed five times with PBS and then incubated with Streptavidin-ALP in PBS plus 0.5% FCS for 1 hour. Plates were washed five times and then developed with filtered BCIP/NBT substrate solution. Flushing the wells with water stopped color development. The plates were air-dried and the number of spot forming cells was determined by subtracting the mean number of spots counted in negative controls wells (n = 3) from the spots counted in each well. The magnitude of the response was determined as the sum of the spots counted per 6×10^5^ PBMC.

### Measuring CHIKV-specific B cell responses

PBMC were stained with surface antibodies directed against CD20 (Beckman Coulter), IgD (Southern Biotech) and CD27 (Biolegend, San Diego, CA) to delineate naive, marginal zone-like and memory B cells. The cells were then fixed and the nuclear membrane permeabilized as per manufacturer's recommendation before addition of Ki67 (BD Pharmingen, San Diego, CA) specific antibodies. Samples were acquired using the LSRII instrument (BD bioscience, San Jose, CA) and data analyzed by FlowJo (TreeStar, Ashland, OR).

Antiviral IgG levels were measured in circulating plasma using a standard ELISA assay using plates coated with CHIKV lysate or CHIKV particle preparations, which reacts with CHIKV Abs. In these experiments, serial three-fold dilutions of plasma were incubated in triplicates CHIKV lysate or virus-coated ELISA plates for 1 hr prior to washing, staining with detection reagents (HRP-anti-IgG) and addition of chromogen substrate to allow for detection and quantitation of bound antibody molecules. Log-log transformation of the linear portion of the curve was then performed, 0.1 OD units was the cut-off point to calculate end point titers. Each plate contained a positive control sample to normalize ELISA titers between assays, and a negative control sample to ensure assay specificity.

### Measuring CHIKV-specific myeloid cell responses in peripheral blood

PBMC were stained with surface antibodies directed against CD3, CD11c, CD14, CD20, CD123 and DR to delineate plasmacytoid dendritic cells, myeloid dendritic cells, DR+ lineage negative other DCs and monocyte/macrophages. Samples were acquired using the LSRII instrument (BD Bioscience, San Jose, CA) and data analyzed by FlowJo (TreeStar, Ashland, OR).

### Measuring bioactive IFN production in plasma

Blood plasma (100 µl) obtained from adult and aged CHIKV infected Rhesus macaques (3 dpi) was UV inactivated (3× for 30 seconds at 600 µJ). Plasma was added to rhesus macaques fibroblasts cultured in 24-well plates for 24 hr. Recombinant universal Type-1 IFN (RND Systems; 0.1, 1, 10 units/ml) treated cells were used as positive controls and untreated cells were used as a negative control. Total RNA was isolated from the cells using the Trizol method (Life Technologies). Quantitiative RT-PCR was used to measure Interferon-stimulated gene (ISG) expression using primers and probes specific for Mx-1 and ISG-56. cDNA was generated using Superscript III (Life Technologies) and analyzed on an ABI StepOne Real-Time PCR system and normalized to L32. Gene amplicons served as quantification standards (limit of detection ≤100 copies/gene). ISG expression levels for each sample were expressed as fold-change by comparison to untreated cells. Data were analyzed by Prism Graph Pad software.

### Statistical analysis

Data were analyzed using Prism Graph Pad software using Two-way ANOVA, no post-test corrections were carried out due to the small sample size.

## Results

### CHIKV infection results in higher and protracted viral loads in aged animals

**Figure 1:**
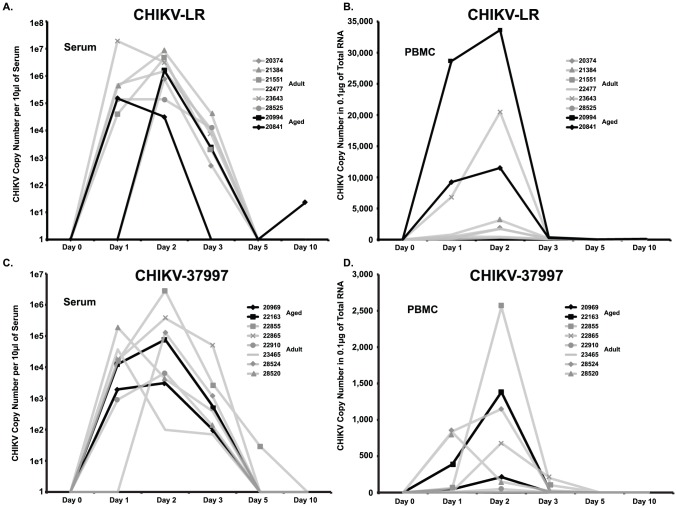
CHIKV viremia in blood peaks 1 to 2 days post infection of Rhesus macaques. CHIKV load in plasma and PBMC was quantified by qRT-PCR using virus specific primers and probes. A and C) Virus was detected in RNA prepared from 10 µl of plasma using ZR Viral RNA extraction kit (Zymol). B and D) Virus was detected in 0.1 µg of total RNA prepared with Trizol from 1×10^6^ PBMC.

Recent outbreak strains of Chikungunya virus contain an adaptive mutation in the E1-protein that facilitates transmission via *Ae. albopictus* (Asian Tiger) mosquitoes as well as increased virulence. We sought to compare CHIKV-LR virus disease progression and host immune responses infection with that of a West African reference strain (37997) to determine whether CHIKV-LR was also more pathogenic in non-human primates. We also explored the impact of aging on virus replication and immune response to both of these strains. We first performed a titration experiment to determine the effects of virus strain and inoculation dosage on *in vivo* CHIKV replication and pathogenesis. Two cohorts of adult rhesus macaques were infected intravenously with either CHIKV-LR or CHIKV-37997. Each cohort consisted of 3 groups of 2 animals each that received 1×10^7^, 1×10^8^ or 1×10^9^ plaque forming units of either strain and animals were observed daily for signs of disease. Animals developed lymphadenopathy 7–14 dpi as well as fever between 3–7 dpi. A rash was observed on the chest of some of the animals at 7 dpi (data not shown). We compared viral loads in peripheral blood mononuclear cells (PBMC) and plasma at 0, 1, 2, 3, 5, and 10 dpi using qRT-PCR (Figure 1). In each cohort viral loads were detectable by 2 dpi and viremia was resolved by 5 dpi. Interestingly, the inoculation dose had no detectable impact on peak viremia or on the kinetics of viral replication (Figure 1). We therefore treated all animals infected with a given strain as one cohort for the remainder of our analysis. While we did not detect statistically significant differences in peak plasma virus levels between CHIKV-LR and CHIKV-37997, average viral loads were nearly 10-fold higher in CHIKV-LR infected animals (5.7×10^6^ vs. 5.9×10^5^ RNA genomic copies per 10 µl of plasma, respectively; p = 0.15). CHIKV RNA levels were very low in PBMC but as described for plasma were slightly higher in CHIKV-LR infected animals (4,650 vs. 878 RNA genomic copies per 0.1 µg of total RNA; respectively, p = 0.29 (Figure 1B vs. 1D).

To investigate whether advanced age is associated with more severe CHIK disease, we infected 4 aged animals (>17 years of age) with 1×10^9^ pfu CHIKV-LR (n = 2) or CHIKV-37997 (n = 2). We compared peak viral loads and the kinetics of viral replication between the aged and adult cohorts (Figure 1). The kinetics of viremia were comparable in both young and aged animals for both virus strains. However, while no virus was detected after 5 dpi in adult animals, one of the aged CHIKV-LR animals had detectable persistent viral RNA in plasma at 10 dpi (Figure 1A; Animal #20841). Levels of virus detected in the PBMC of the aged CHIKV-LR infected animals tended to be higher compared to adult animals or aged animals infected with CHIKV-37997 (Figure 1B &D).

**Figure 2:**
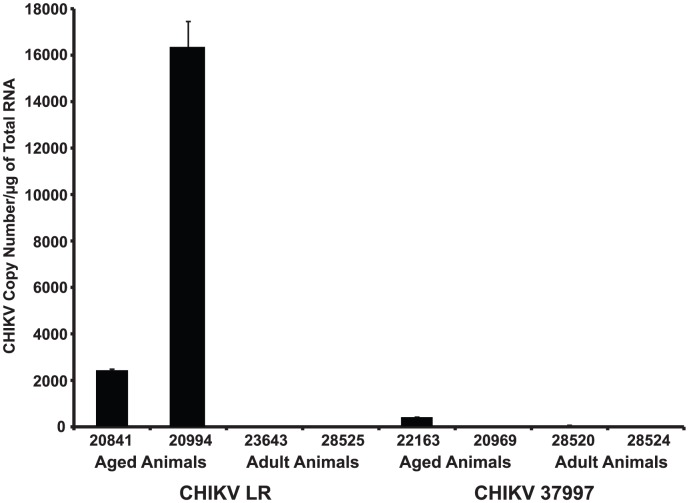
CHIKV-LR persists in spleen of aged Rhesus macaques. CHIKV load was quantified in spleen tissue at necropsy (35 dpi) by virus-specific qRT-PCR from total RNA samples prepared using Trizol.

All animals were euthanized at 35–42 dpi, at which point, blood, spleen, lymph nodes and joint tissues were harvested to determine viral loads. At the time of necropsy, we observed no gross joint inflammation in any of the animals. In addition, we detected no virus in tissues harvested from adult animals at 35 dpi. However, viral RNA was present in spleen of aged CHIKV-LR infected animals but not in aged animals infected with CHIKV-37997 (Figure 2). Interestingly, aged animal #20994 infected with CHIKV-LR had both the highest levels of PBMC associated virus and highest level of spleen-associated virus compared to all other infected animals. These findings suggest that CHIKV-LR can persist in aged animals and is potentially more virulent than 37997. The higher PBMC viral loads in CHIKV-LR infected aged animals as well as the persistence of viral RNA in the spleen could be due to increased viral replication and/or an ineffective immune response.

### T cell responses are reduced in aged animals following CHIKV infection

**Figure 3:**
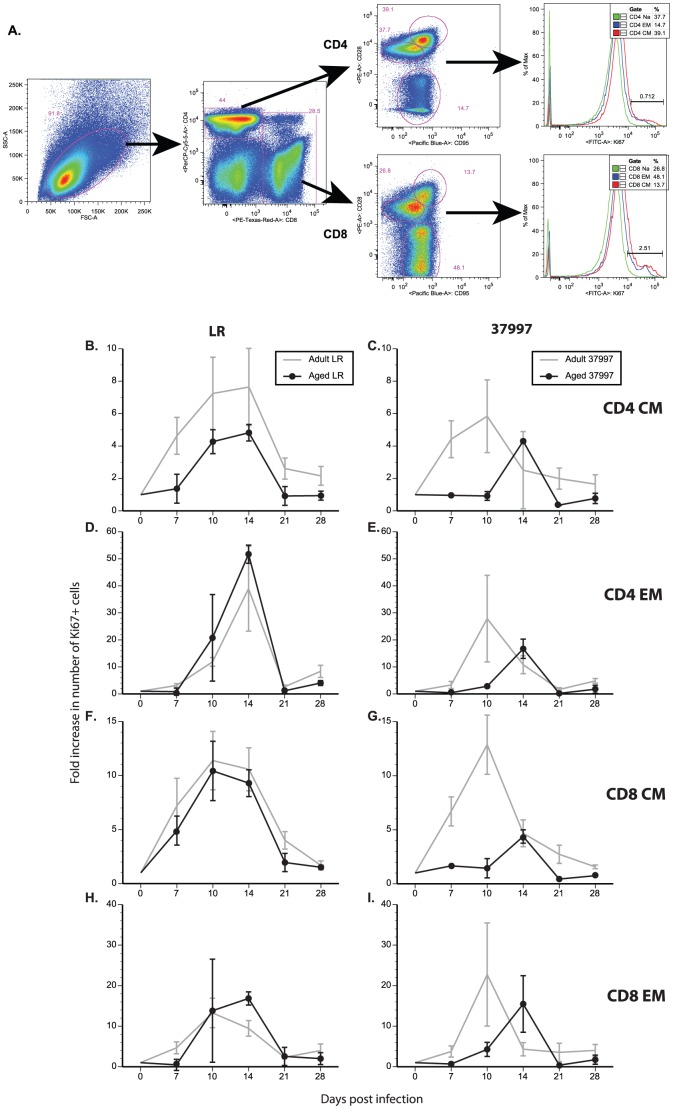
Aged Rhesus macaques have lower T cell responses following CHIKV infection. T-cell proliferative burst was measured following CHIKV infection. PBMC were stained with antibodies directed against CD4, CD8, CD28, CD95 and Ki67 (as shown in panel A). Fold increase in number of Ki67+ cells was calculated for each time point. Cell populations were subdivided: B & C) CD4+ Central memory (CM) T-cells; D & E) CD4+ Effector memory (EM) T-cells; F & G) CD8+ CM; H & I) CD8+ EM for animals infected with CHIKV-LR (panels B, D, F, H) or CHIKV-37997 (panels C, E, G, I). N = 6 for adult and N = 2 for aged animals.

Next, we investigated the impact of age on the adaptive immune response to CHIKV. One of the hallmarks of the immune response against a viral infection is rapid and robust proliferation of antigen specific lymphocytes. We therefore compared the kinetics and magnitude of the T cell proliferative burst in adult and aged animals by determining the frequency of T cells expressing the nuclear protein Ki67, which is induced during cell cycle [25]. Peripheral blood mononuclear cells isolated at different time points after infection were first stained with antibodies directed against CD4, CD8, CD28 and CD95 in order to delineate naïve, central (CM) and effector memory (EM) T cell populations as shown in Figure 3A and previously described [25]. The cells were permeabilized, stained with antibodies directed against Ki67, and analyzed by flow cytometry (FCM).

Proliferation of CD4 CM cells was evident by 7 dpi in both CHIKV-LR and CHIKV-37997-infected adult animals (Figure 3B & C). The frequency of Ki67+ CD4 CM T cells peaked at 10 dpi and remained elevated in CHIKV-LR infected animals until 14 dpi before returning to baseline at 28 dpi (Figure 3B). On the other hand, after peaking at 10 dpi, the frequency of CD4 CM Ki67+ T cells began to decrease in CHIKV-37997 infected animals, returning to baseline at 21 dpi (Figure 3C). However, the CD4 CM T cell proliferative burst was delayed in aged animals. Specifically, CD4 CM proliferation was not initiated until 10 dpi in aged CHIKV-LR infected animals and 14 dpi in aged CHIKV-37997 animals (Figure 3B & C; p = 0.037 and p = 0.005, respectively). Moreover, the CD4 CM proliferative burst was sustained longer in aged animals infected with the LR strain compared to 37997. Overall, the frequency of proliferating CD4 CM T cells was lower in aged animals compared to adults, and this difference was most noticeable in CHIKV-37997 infected animals (Figure 3B & C). CD4 EM T cell proliferation was detected 10 dpi and peaked 14 dpi in LR infected young and aged animals before returning to baseline at 21 dpi (Figure 3D). In 37997 infected adult animals, CD4 EM T cell proliferation was detected and peaked10 dpi returning to baseline by 21 dpi (Figure 3E). As described for CD4 CM, CD4 EM T cell proliferation was delayed in aged animals infected with CHIKV-37997 the peak occurring 14 dpi and returning to baseline levels 21 dpi (Figure 3E; p = 0.055). In summary, proliferation of the CD4 CM subset preceded that of the CD4 EM subset. In addition, CD4 T cell proliferative burst was greater in animals infected with the LR strain compared to 37997 and aged animals infected with 37997 generated a smaller CD4 T cell proliferative response than adult animals.

Similar patterns of proliferation were observed in the CD8 T cell subsets. Proliferation within the CD8 CM subset was detected in all LR-infected animals at 7 dpi (Figure 3F & G). Proliferation peaked at 10 dpi and was sustained in both adult and aged animals infected with the CHIKV-LR strain through 14 dpi, returning to baseline by 21 dpi (Figure 3F). In contrast, CD8 CM proliferative burst was shorter in adult animals infected with 37997 peaking 10 dpi and declining 14 dpi (Figure 3G). Aged animals infected with 37997 exhibited a delayed and blunted CD8 CM proliferative response compared to adult animals (Figure 3G; p = 0.061). Proliferation within the CD8 EM subset was detected 10 dpi, remained stable until 14 dpi returning to baseline 21 dpi in CHIKV LR-infected animals (Figure 3H). Adult and aged CHIKV-LR infected animals generated comparable CD8 EM proliferative bursts. CD8 EM in 37997-infected adult animals peaked 10 dpi before returning to baseline 14 dpi. As described for all other subsets, CD8 EM proliferation was delayed in 37997-infected aged animals (p = 0.126). In summary, aged animals infected with CHIKV-LR showed a comparable CD8 T cell proliferative burst following viral infection compared to adult animals. In contrast, T cell proliferation in aged animals infected with CHIKV-37997 was delayed and blunted compared to that of adult animals.

**Figure 4:**
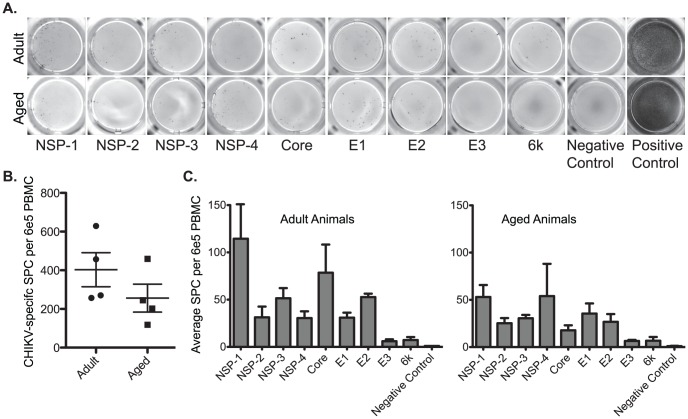
Magnitude and breadth of anti-CHIKV T cell responses is reduced in aged Rhesus macaques. IFN-γ ELISPOT assays were used to quantify anti-CHIKV T cell responses in peripheral blood lymphocytes at 35 dpi in adult and aged animals (n = 4). In triplicate wells, PBMC (2×10^5^) were incubated overnight with overlapping peptides that corresponded to each of the 9 CHIKV proteins (NSP-1-4, Core, E1-3 and 6k). PMA/ionomycin stimulation was used as a positive control and medium plus DMSO was used as a negative control. A) Representative wells from IFN-γ ELISPOT assay performed with PBMC from adult and aged animals. B) Average cumulative anti-CHIKV responses to all viral proteins were compared for adult vs. aged Rhesus macaques (n = 4). C) Average response to individual CHIKV proteins (n = 4).

To further characterize the impact of age on T cell response to CHIKV infection *in vivo*, we used IFN-γ ELISPOT assays to determine the frequency of CHIKV-specific T cells in PBMC. A complete overlapping peptide library to CHIKV was subdivided into peptides for each of the 9 proteins (NSP-1-4, Core, E1-3 and 6k). PBMC collected at 35 dpi were incubated with peptides and the mean numbers of spot forming units [26] per 6×10^5^ PBMC were determined for four adult and four aged animals. A representative example of the data is presented in Figure 4A and the data summary is provided in Figure 4B. T cell response to CHIKV antigens was quite broad targeting both structural and nonstructural proteins. The CHKV-specific T cell response in adult animals was higher compared to the aged animals (403±88 vs. 256±73, respectively; n = 4). Similarly, the breadth of the anti-CHIKV T cell response to any given viral protein differed slightly between adult and aged animals (Figure 4C). Frequencies of T cells specific for NS1 dominate the T cell response in both adult and aged animals, whereas the response to core protein was the second largest in adult animals, NSP4 was the second largest target for aged T cells. These data indicate that advanced age results in reduced frequency and altered breadth of anti-CHIKV T cells response.

### Aged animals generate reduced B cell responses following CHIKV infection

**Figure 5:**
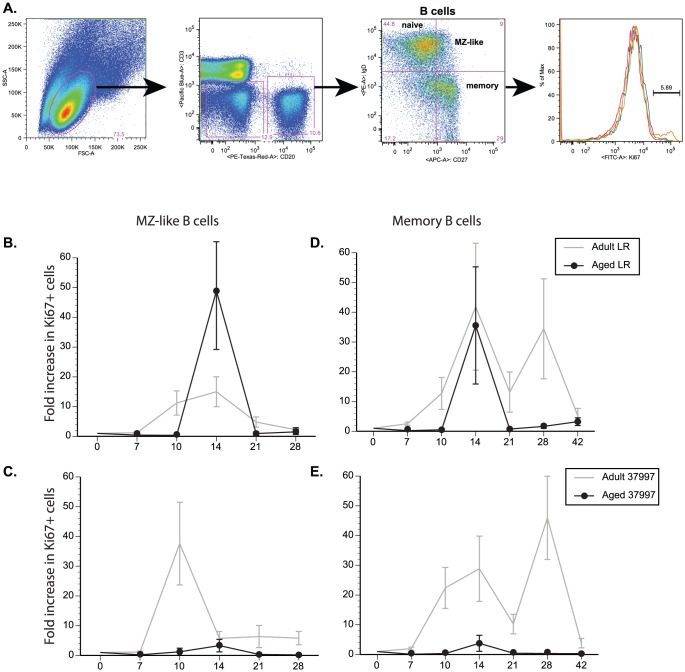
Aged Rhesus macaques have a delayed and lower B cell proliferative response following CHIKV infection. Measurement of B-cell proliferative burst following viral infection. PBMC were stained with antibodies directed against CD20, CD27, IgD and Ki67 (as shown in panel A) and subdivided into (B & C) Marginal Zone (MZ)-like B cells and (D & E) memory B cells. Fold increase in number of Ki67+ B cells was calculated at each time point. N = 6 for adult and N = 2 for aged animals.

We next assessed the B cell response by measuring the kinetics and magnitude of the B cell proliferative response as well as IgG production following infection. B cells can be subdivided into three subsets based on the expression of CD27 and IgD as shown in Figure 5A and previously described [25]: naïve (Na, IgD+, CD27−), marginal zone like (MZ-like, IgD+, CD27+) and memory B cells (IgD−, CD27+). MZ-like B cell proliferation in adult animals infected with CHIKV-LR was detected at 10 dpi, peaked around 14 dpi and returned to baseline by 21 dpi (Figure 5B). MZ-like B cell proliferation in aged animals was not detected until 14 dpi (Figure 5B). Proliferation in the memory B cell subset was detected at 14 dpi in adult and aged CHIKV-LR-infected animals (Figure 5D). As described for T cell subsets, B cell proliferation was delayed and reduced in CHIKV-37997-infected aged animals compared to adult (Figure 5C & E). This difference was most noticeable for MZ-like B cells (p = 0.074). Of note, memory B cell proliferation seemed biphasic with detection of a second peak in adult animals infected with both virus strains at 28 dpi that was absent in the aged animals. This second proliferative peak could be indicative of MZ-like B cells acquiring memory phenotype and continuing to proliferate in adult animals. The lack of this second proliferative burst in memory B cells in aged animals is likely another indication of the failures of an aged immune system.

**Figure 6:**
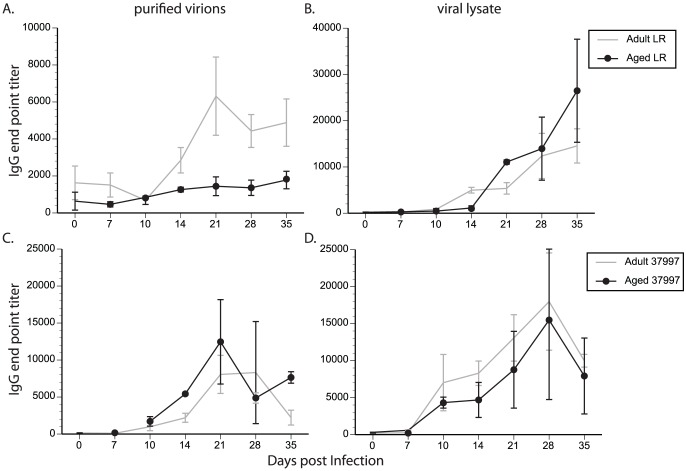
Aged Rhesus macaques have reduced anti-virion antibody titers compared to adult animals following CHIKV infection. The production of anti-CHIKV antibodies in aged Rhesus macaques was compared to adult animals. CHIKV-specific endpoint IgG titers were measured using standard ELISA detecting either CHIKV purified virions (A & C) or CHIKV infected cell lysates (B & D) as the antigenic source. N = 6 for adult and N = 2 for aged animals. Aged animals have lower antibody titers to whole virions compared to adult animals, whereas the production of IgG responses to antigen present in CHIKV-infected cellular lysates were not age dependent.

To further characterize B cell response, we measure IgG end point titers by ELISA using 96-well plates coated with either purified virus or cellular lysates produced from infected Vero cells. When using purified virions as the antigen source, anti-CHIKV IgGs were detected at 14 dpi and peaked near 21 dpi in LR-infected adult animals (Figure 6A). The IgG response was significantly reduced in aged animals (p<0.05 for days 21, 28 and 35). When using viral lysate as antigen, IgG titers were first detected 14 dpi, continued to increase until 35 dpi and were comparable between adult and aged animals (Figure 6B). Interestingly, the peak IgG titers to viral lysate were 3-fold higher than those to whole virions (end point titer of 14545 vs. 4881, respectively). Similar kinetics were observed for the IgG response to CHIKV-37997 in adult animals (Figure 6C & D). The IgG titers were also higher in response to lysate antigen compared to virions (4-fold) (Figure 6C & D). However, in CHIKV-37997-infected aged animals, peak IgG titers to virions or lysate were comparable between aged and adult animals (Figure 6C & D). These data taken together indicate that IgG response against viral determinants is reduced in LR-infected aged animals, possibly contributing to viral persistence in these animals. Further experimentation will be required to determine whether there are differences in specific antibody binding epitopes or avidity of antibodies that bind CHIKV between the aged and adult RM [23], [24].

### Alteration of monocyte and dendritic cell populations following CHIKV infection is age-dependent

**Figure 7:**
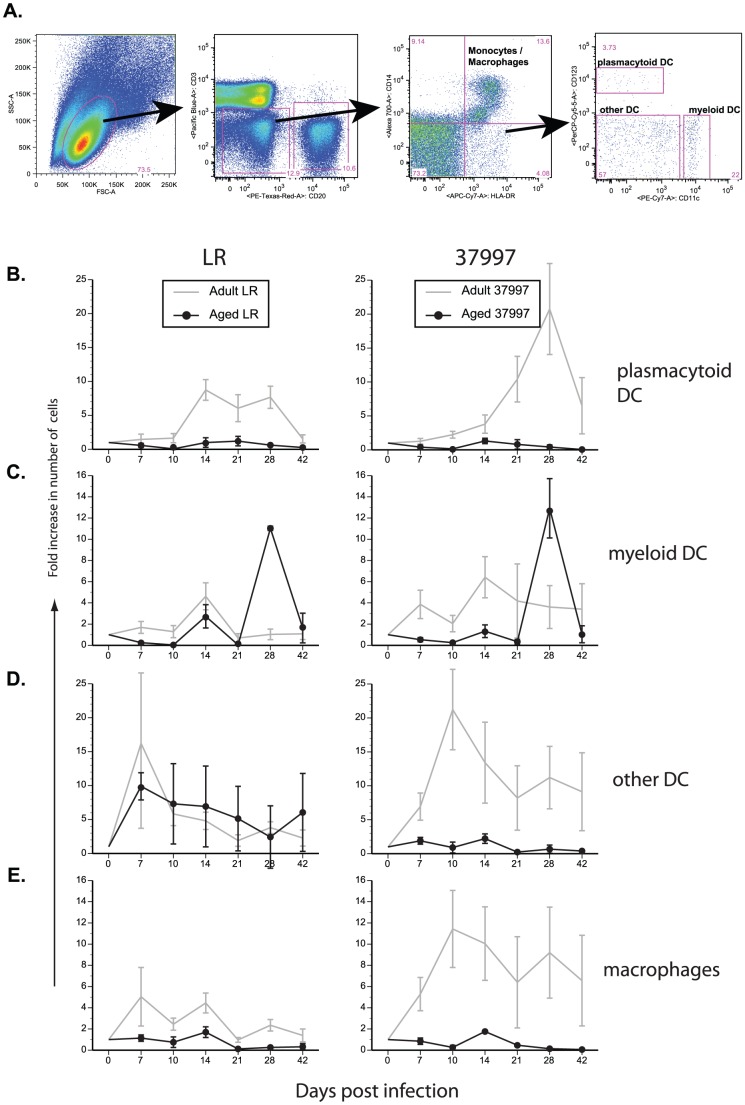
Macrophage and dendritic cell population changes following CHIKV infection observed in adult animals are reduced in aged Rhesus macaques. Measurement of dendritic cell and monocyte/macrophage populations following CHIKV infection. PBMC were stained with antibodies directed against CD3, CD11c, CD14, CD20, CD123 and HLA-DR (shown in panel A) and subdivided into plasmacytoid DCs (B); myeloid DCs (C); non-P/non-M DC's (D); monocyte/macrophages (E). Fold increase in numbers of cells was calculated. N = 6 for adult and N = 2 for aged animals.

Given the proposed role for monocyte/macrophages in CHIKV pathogenesis [27] and formation of CHIKV immune response, we characterized the effects of CHIKV infection on myeloid cell populations in peripheral blood. For this assay, total PBMC were stained with antibody panels allowing separation of myeloid cells into 4 subpopulations: plasmacytoid dendritic cells (pDC: CD3−, CD11c−, CD14−, CD20−, CD123+ and DR+); myeloid dendritic cells (mDC: CD3−, CD11c+, CD14−, CD20−, CD123− and DR+); non-plasmacytoid/non-myeloid dendritic cells (other DC: CD3−, CD11c−, CD14−, CD20−, CD123− and DR+); and monocyte/macrophages (CD3−, CD11c−, CD14+, CD20−, CD123− and DR+) as depicted in Figure 7A. The frequency of all DC subsets as well as monocyte/macrophages, increased in the peripheral blood of adult animals after CHIKV infection (Figure 7B–E). Of note, frequency of pDCs increased ∼14 dpi only in adult animals (Figure 7B; p = 0.017 and p = 0.164 for LR vs. 37997 at 14 dpi respectively). This overall increase appeared more dramatic following infection with 37997 compared to LR strain. Frequency of mDCs increased in LR infected adult and aged animals 14 dpi before returning to baseline (Figure 7C). In 37997 infected adult animals, mDC frequencies increased 7 dpi and remained fairly stable whereas aged animals did not experience any changes in mDC numbers. Frequency of other DC increased following infection with CHIKV-LR strain reaching comparable peak levels 7 dpi in both adult and aged animals before returning slowly to baseline levels 42 dpi (Figure 7D). In contrast, infection with 37997 induced an increase in the frequency of other DCs only in adult animals (p = 0.029). Lastly, frequencies of monocyte/macrophages increased only in adult animals infected with both CHIKV strains (Figure 7E; LR p = 0.019 and 37997 p = 0.048). As described for pDCs and other DCs, this increase was more dramatic following 37997 than LR infection.

**Figure 8:**
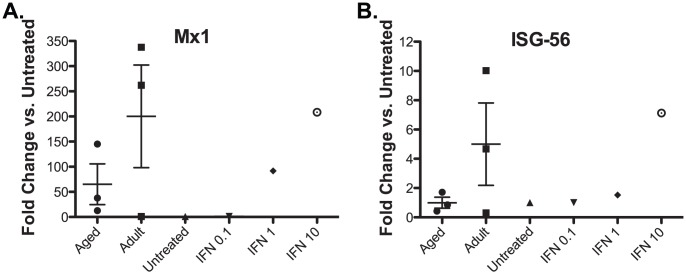
Plasma from aged Rhesus macaques have lower levels of bioactive IFN compared to adult animals. Plasma samples from adult (Animal #22855, 21551, 20374) and aged (Animal #20969, 22163, 20994) RM at 3 days post infection with CHIKV were serially diluted and used to stimulate Rhesus fibroblasts for 24 hours. Recombinant universal Type-1 IFN stimulated cells (0.1, 1, 10 units/ml) were used as positive controls and untreated cells were used as a negative control. Then total RNA was prepared with Trizol and IFN stimulated genes Mx1 (A) and ISG56 (B) were quantified by qRT-PCR using gene-specific primers. Fold change is determined as the ratio of gene expression treated vs. untreated control samples.

To determine whether the innate immune response was also affected by age following CHIKV infection, we measured plasma levels of bioactive IFN. For this experiment, rhesus macaque fibroblasts were treated with UV inactivated plasma from CHIKV infected aged or adult animals at 3 dpi. Interferon stimulated gene (ISG) expression was determined by quantitative RT-PCR using specific primers. We detected a trend towards increased ISG expression in fibroblasts treated with plasma from the adult animals indicating that the levels of bioactive IFN was higher in those samples but this difference did not reach statistical significance (Figure 8).

## Discussion

Chikungunya virus is a re-emerging Alphavirus that causes debilitating arthralgia. The 2006 outbreak on the island of La Reunion involved a newly emerged CHIKV strain. This strain exhibits both an increased vector range making more widespread transmission highly likely but also more severe virus-induced disease. Ultimately these phenomena demonstrate that CHIKV is an adaptable pathogen capable of rapidly expanding its ecological niche and potentially emerging globally. Moreover, this new strain was associated with increased mortality in the elderly and newborns. Unfortunately, the host response to CHIKV infection and associated determinants of disease remain poorly understood. Characterization of the immune response following CHIKV infection in humans is hampered by the occurrence of symptoms at least a week after infection as well as severity of disease that can differ dramatically between patients, which both influence the time of presentation in the clinic. Thus, novel models that recapitulate human CHIKV disease are needed to facilitate the identification of the immune correlates of protection against CHIKV infection, and for evaluation of therapeutics and vaccines. Recently studies in Cynomolgus monkeys have shown that infection of non-human primates results in similar pathophysiology as that described for humans with the involvement of several joints, but the host immune responses were not evaluated [22]. Therefore, we compared the immune response following infection with the recent outbreak strain CHIKV-LR to the West African strain 37997 in adult and aged rhesus macaques.

This is the first report describing the impact of age on CHIKV disease and immune response in non-human primates. Our results show that although viremia is detected within 24 hpi in both adult and aged animals, LR infection resulted in slightly higher peak viremia. Interestingly, at necropsy (35 dpi) we were able to detect persistent viral RNA in spleens harvested from aged but not adult animals infected with the LR strain suggesting that aged animals were unable to fully clear viral infection. One possible explanation for persistent viral RNA in aged animals is a reduced immune response to CHIKV. Therefore, we characterized the innate and adaptive immune responses following CHIKV infection. Following infection with CHIKV-LR, adult and aged rhesus macaques generated comparable T and B cell proliferative responses, similar IgG antibodies to viral lysate but lower IgG antibodies to whole virions. In contrast, aged animals infected with 37997 generated weaker T cell and B cell proliferative responses (delayed and reduced) compared to adult animals, but the IgG responses were comparable. We also measured frequency of CHIKV-specific T cells using an overlapping peptide library for the entire CHIKV proteome. Overall CHIKV-specific T cell responses were lower in aged animals and the immunogenicity profiles differed. Aged animals also failed to exhibit an increase in the frequency of myeloid innate immune cells. This failure could be mediated by a defect in innate immune cell mobilization or proliferation. Lastly, production of bioactive Type 1 IFN was lower in the plasma of the aged animals at 3 dpi. Taken together, our findings suggest that aged animals have impaired ability to mount an effective immune response against CHIKV infection.

Aging results in a general decline in immune function, commonly known as immune senescence, which contributes to the increased susceptibility to pathogens and cancer. Defects associated with immune senescence have been shown in both innate and adaptive processes. In the current report we found a reduction in innate immune responses in the aged animals infected with CHIKV compared to adult animals. Importantly, these responses were observed in animals infected with either the LR or 37997 strains. Of note, aged animals do not exhibit an increase in the frequency of pDC. Given that pDCs play a critical role in the antiviral response through the production of Type 1 IFN [28], the results presented in this manuscript suggest that pDCs and production of type 1 IFN may contribute to the successful control of CHIKV viremia. Previous studies have shown a decrease in circulating pDC numbers with increasing age [29], [30]. The data presented here suggest additional defects in pDC mobilization. We observed a corresponding reduction in the production of plasma levels of bioactive Type 1 IFN in aged animals. Both clinical data and rodent studies using IFNαR-/- and STAT1-/- mice demonstrate a critical role for type I IFN in CHIKV clearance [20], [31]. Others have demonstrated an age-related decrease in IFN response through STAT1, IRF1 and IRF7 signaling following infection with West Nile Virus [32], [33]. Other cell types of the innate immune response such as macrophages and myeloid DCs play an important role in antigen presentation and the initiation of the adaptive immune response. Age-related defects in macrophage and DC antigen presentation and migration reduce their ability to prime naïve T cell responses to novel pathogens [34], [35]. Aged animals infected with CHIKV did not show an increase in monocyte/macrophage and mDCs following CHIKV infection. This defect could in part explain the reduced T cell proliferation observed in 37997 infected animals

In line with decreased magnitude of the T cell proliferative burst, we found reduced frequency of CHIKV-specific T cells in the aged animals by IFN-γ ELISPOT. Interestingly, the immunodominance profile also differed between adult and aged animals. Similarly, we observed a decreased IgG antibody response to whole virions. However, IgG titers measured using plates coated with viral lysate were higher than those measured using purified virions suggesting that some of the antibody response is directed against viral antigens that are not included in the virion. Collectively, these findings indicate that the aged immune system is incapable of mounting a broad and effective adaptive immune response to CHIKV.

In summary, we present data indicating that the recent outbreak strain CHIKV-LR replicated, on average, to higher levels compared to the CHIKV strain 37997. While we did not observe CHIKV-associated joint inflammation or disease for either virus in these animals at 35 dpi, we did identify the spleen as a potential site of CHIKV persistence in aged animals. This effect was limited to CHIKV-LR and did not occur in monkeys infected with CHIKV-37997, which is in line with the increased severity of CHIKV-LR disease. We have also identified a number of differences in the ability of adult vs. aged animals to respond to CHIKV infection, with a general decline in both the innate and adaptive immune responses that could explain the increased disease severity observed in older individuals.
